# Ultrafast photocurrents at the surface of the three-dimensional topological insulator Bi_2_Se_3_

**DOI:** 10.1038/ncomms13259

**Published:** 2016-10-31

**Authors:** Lukas Braun, Gregor Mussler, Andrzej Hruban, Marcin Konczykowski, Thomas Schumann, Martin Wolf, Markus Münzenberg, Luca Perfetti, Tobias Kampfrath

**Affiliations:** 1 Fritz Haber Institute of the Max Planck Society, 14195 Berlin, Germany; 2 PGI-9 and JARA-FIT, Forschungszentrum Jülich, 52425 Jülich, Germany; 3 Institute of Electronic Materials Technology, 01-919 Warsaw, Poland; 4 Laboratoire des Solides Irradiés, Ecole Polytechnique, CNRS, CEA, Université Paris-Saclay, 91128 Palaiseau cedex, France; 5 Institut für Physik, Ernst-Moritz-Arndt-Universität Greifswald, 17489 Greifswald, Germany

## Abstract

Three-dimensional topological insulators are fascinating materials with insulating bulk yet metallic surfaces that host highly mobile charge carriers with locked spin and momentum. Remarkably, surface currents with tunable direction and magnitude can be launched with tailored light beams. To better understand the underlying mechanisms, the current dynamics need to be resolved on the timescale of elementary scattering events (∼10 fs). Here, we excite and measure photocurrents in the model topological insulator Bi_2_Se_3_ with a time resolution of 20 fs by sampling the concomitantly emitted broadband terahertz (THz) electromagnetic field from 0.3 to 40 THz. Strikingly, the surface current response is dominated by an ultrafast charge transfer along the Se–Bi bonds. In contrast, photon-helicity-dependent photocurrents are found to be orders of magnitude smaller than expected from generation scenarios based on asymmetric depopulation of the Dirac cone. Our findings are of direct relevance for broadband optoelectronic devices based on topological-insulator surface currents.

## Abstract (web summary)

Surface currents in topological insulators can be controlled by light, but the underlying mechanisms are not well understood. Here, Braun *et al*. report an ultrafast shift photocurrent at the surface of Ca-doped Bi_2_Se_3_, whereas injection currents are much smaller than expected from asymmetric depopulation of the Dirac cone.

## Graphical abstract

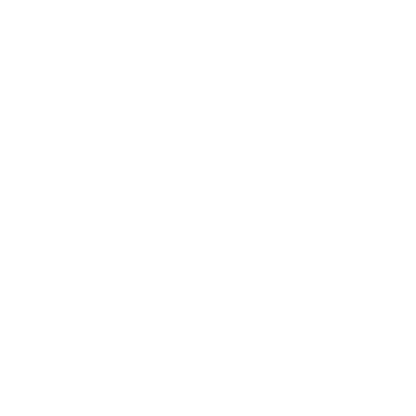

Many efforts in current solid-state research aim at pushing the speed of electronic devices from the gigahertz to the terahertz (1 THz=10^12^ Hz) range^1^ and at extending their functionalities by the spin of the electron^2^. In these respects, three-dimensional topological insulators (TIs) are a highly promising material class. Although having an insulating bulk, their surface is metallic due to a band inversion that is topologically protected against external perturbations. Bi_2_Se_3_ is a model TI^3^ as its surface features a single pair of linear Dirac-type electronic energy bands^4^ with spin-velocity locking and forbidden 180° backscattering^5^. These properties are ideal prerequisites to induce large spin polarizations by means of surface currents.

Part of this considerable potential was demonstrated by recent works, which reported the exciting possibility of launching TI surface currents by simply illuminating the sample with light^6^,^7^,^8^,^9^,^10^,^11^,^12^,^13^. The direction of the photocurrent could be controlled through the polarization state of the incident light beam. The assignment to a surface process was supported by picosecond time-of-flight measurements^13^ showing that the photoinduced carriers were propagating at a speed comparable to the band velocity of the Dirac states. There is, however, still an intense debate about mechanisms leading to TI surface photocurrents. Scenarios based on asymmetric depopulation of the Dirac cone^6^ transitions into other higher-lying cones^13^, and asymmetric scattering of electrons^8^ have been proposed. To directly resolve the generation of TI surface photocurrents, we need to boost the time resolution of the experiment from so far as ∼250 fs and longer^9^,^10^,^11^,^12^,^13^ to the scale of elementary scattering events, which can be shorter than 10 fs.

Here, we use ultrabroadband terahertz (THz) emission spectroscopy^14^,^15^,^16^ from 0.3 to 40 THz to probe the ultrafast evolution of photocurrents in the Ca-doped model TI Bi_2_Se_3_ with unprecedented time resolution. On the basis of an analysis of their temporal structure and symmetry, we identify distinct current sources. First, a slow drift current of photoinduced bulk charge carriers along the TI surface field is found. Second, we observe that currents depending on the pump helicity are orders of magnitude smaller than expected from the photocurrent generation scenario based on asymmetric depopulation of the Dirac cone^6^. This remarkable result suggests a strong mutual cancellation of the contributions of the various optical transitions, much reduced matrix elements for surface-to-bulk transitions and/or relatively small pump-induced changes in the electron band velocity. Finally, for the first time, we observe a new type of photocurrent, a surface shift current, which originates from an instantaneous displacement of electron density along the Se–Bi bond. This charge transfer is localized in a surface region of ∼2 nm thickness, the natural confinement scale of topological surface states. Its relaxation time of 22 fs provides the timescale on which the optically excited surface carriers relax to an isotropic distribution. In terms of applications, the instantaneous electric field generated by the shift current could be used to drive highly spin-polarized THz electric currents at the TI surface along an easily tunable direction.

## Results

### Ultrafast photocurrent amperemeter

**Figure 1:**
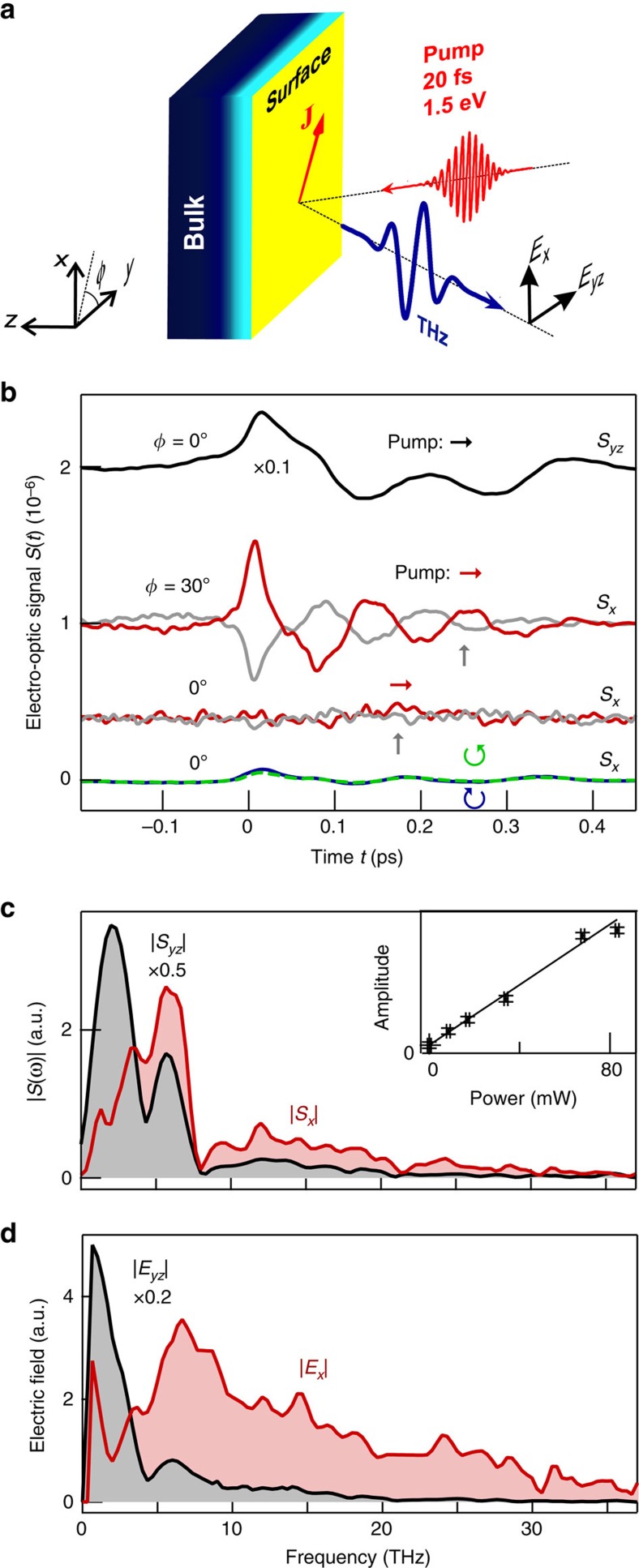
THz emission experiment and raw data. (**a**) Schematic of the ultrafast photocurrent amperemeter. A Bi_2_Se_3_ crystal is excited by a femtosecond laser pulse, resulting in a photocurrent burst and, consequently, emission of a THz electromagnetic pulse. Measurement of the transient THz electric field components *E*_*x*_(*t*) and *E*_*yz*_(*t*) by electro-optic sampling provides access to the sheet current **J**(*t*) flowing inside the sample. (**b**) Typical *x*- and *yz*-polarized THz electro-optic signals *S*_*x*_ and *S*_*yz*_ measured for various settings of pump polarization and sample azimuth *ϕ*. The signals are offset for clarity. (**c**) Amplitude spectra of the THz signal and (**d**) THz electric field directly behind the sample as extracted from **b**.

A schematic of our experiment is depicted in Fig. 1a. A femtosecond laser pulse is incident on the specimen and launches a transient charge current density **j**(*z*,*t*). This photocurrent, in turn, emits an electromagnetic pulse with transient electric field **E**(*t*), in particular, covering frequencies up to the THz range, as expected from the inverse duration of the femtosecond stimulus. The measurement of **E**(*t*) over a large bandwidth (0.3 to 40 THz) permits extraction of the sheet current density


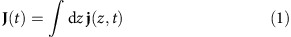


with ultrafast time resolution.

As detailed in the ‘Methods' section, this approach allows us to separately determine the current component *J*_*x*_ directed along the *x* axis and the component *J*_*yz*_, which is a linear combination of the roughly equally weighted Cartesian components *J*_*y*_ and *J*_*z*_ (see Fig. 1a and the ‘Methods' section). By virtue of a generalized Ohm's law, the currents *J*_*x*_ and *J*_*yz*_ are, respectively, connected to the *s*-polarized electric-field component *E*_*x*_ and the perpendicular, *p*-polarized component *E*_*yz*_ directly behind the sample (Fig. 1a). The THz near-fields *E*_*x*_ and *E*_*yz*_ are obtained by measuring the THz far-field using electro-optic sampling, resulting in the electro-optic signals *S*_*x*_ and *S*_*yz*_, respectively (see the ‘Methods' section). THz waveforms are acquired at the shot-noise limit of the setup and for various settings of the pump polarization and sample azimuth *ϕ* (Fig. 1b).

We use this approach to study a freshly cleaved, n-type, Ca-doped Bi_2_Se_3_ single crystal in ambient air (see the ‘Methods' section). While dipolar photocurrents in the inversion-symmetric crystal bulk (space group 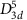) cancel, optical excitation can in principle launch a current at the surface (space group *C*_3*v*_)^17^. The surface region can be thought to consist of the air–crystal interface with locally relaxed lattice structure, which overlaps with the Dirac surface states (thickness of ∼2 nm)^18^,^19^, followed by a space–charge region with bent bulk bands (thickness of tens of nanometres)^20^,^21^. As the sample thickness (>300 μm) is much larger than the pump penetration depth (24 nm; ref. 20), the front but not the back surface of the sample is probed. Further details on sample properties and characterization can be found in the ‘Methods' section.

In what follows, we will show that our broadband current measurements allow us to discriminate different types of photocurrents and their generation in the various surface regions. This goal is achieved by first identifying two dominating components in the THz emission signal using symmetry analysis. Finally, on the basis of their temporal structure and symmetry, the two underlying photocurrent components are assigned to microscopic generation scenarios.

### Raw data

Typical THz electro-optic signal waveforms *S*(*t*) obtained from our Bi_2_Se_3_ sample are shown in Fig. 1b. The THz waveforms depend sensitively on the setting of the THz polarization (*x* versus *yz*), the pump polarization and the sample azimuthal angle *ϕ*. The signal amplitude grows linearly with increasing pump power, without any indication of saturation (inset of Fig. 1c). This behaviour implies that the number of excited carriers is proportional to the incident photon number.

**Figure 2:**
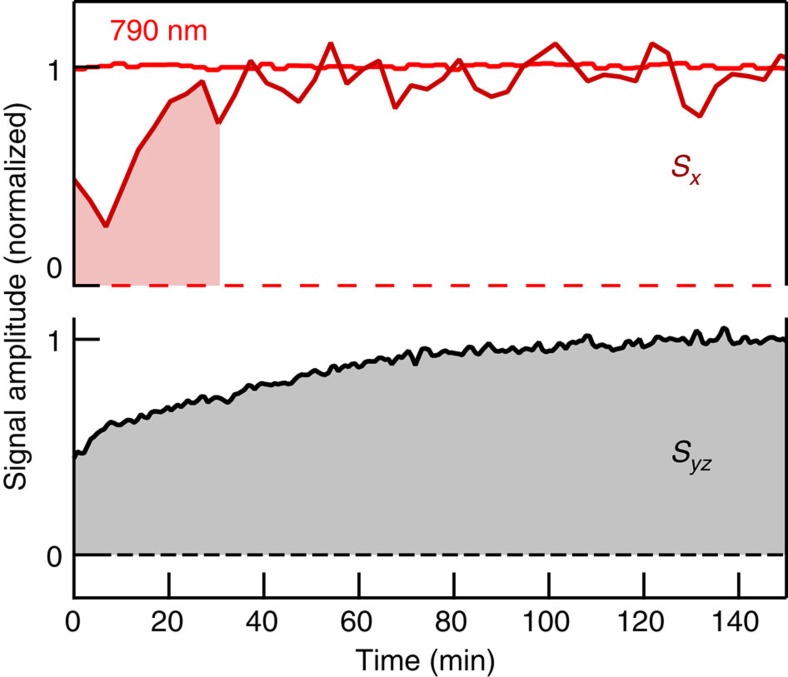
Long-term signal changes. Evolution of the amplitudes of THz signals *S*_*x*_ and *S*_*yz*_ and the reflectance of the 790 nm pump beam following cleaving of the sample. The constant 790 nm signal indicates that no sample damage occurs.

**Figure 3:**
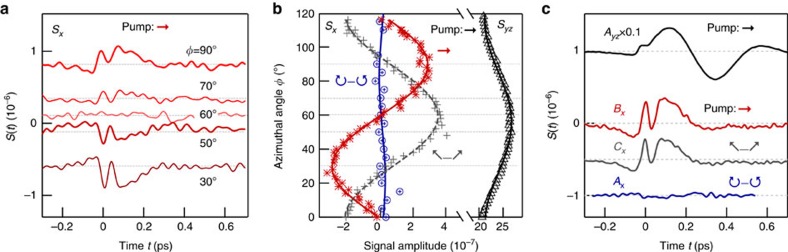
Azimuth dependence and dominant components of THz signal *S*_*x*_. (**a**) THz signal waveforms at various sample azimuth angles *ϕ* and for fixed pump polarization. (**b**) Extracted THz amplitude versus *ϕ* for various pump-polarization settings (→ and differential signals ↖−↗ and 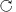
−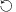). While both *S*_*x*_ and *S*_*yz*_ exhibit a 3*ϕ*-type-dependent component of comparable magnitude, *S*_*yz*_ has a much larger and dominant component independent of *ϕ*. (**c**) Dominant temporal components of signal sets *S*_*yz*_(*t*, *ϕ*) and *S*_*x*_(*t*, *ϕ*) for various pump polarizations, extracted by using equation (2) (see the ‘Methods' section).

As detailed in the following, we make the striking observation that the *x*- and *yz*-polarized components of the emitted THz field (and, thus, *J*_*x*_ and *J*_*yz*_) behave very differently in terms of their magnitude (Fig. 1b), temporal shape (Fig. 1b), their behaviour after sample cleavage (Fig. 2) and their dependence on the sample azimuth *ϕ* (Fig. 3b). First, as seen in Fig. 1b, *S*_*yz*_ exhibits much larger amplitude than *S*_*x*_ but evolves significantly more slowly. Accordingly, the amplitude spectrum of *S*_*x*_ exhibits a larger bandwidth than *S*_*yz*_ (Fig. 1c). This trend becomes even clearer when we apply an inversion procedure to these data to extract the transient THz fields *E*_*x*_ and *E*_*yz*_ directly behind the sample (see the ‘Methods' section). The resulting spectral amplitudes are displayed in Fig. 1d as a function of angular frequency *ω* and show that |*E*_*x*_(*ω*)| is much broader than |*E*_*yz*_(*ω*)|, indicating much faster temporal dynamics. The spectrum exhibits features such as the dips of |*E*_*x*_(*ω*)| at *ω*/2*π*=2 THz and 4 THz whose origin becomes clear further below.

Second, to investigate the impact of surface modification on *S*_*x*_ and *S*_*yz*_, we freshly cleave the sample and subsequently acquire THz signals continuously over >2 h with the sample exposed to air. While the shape of the THz waveforms does not undergo measurable modifications, their global amplitude increases by a factor of ≈2 in the course of time (Fig. 2). Note this rise proceeds within 30 min for *S*_*x*_ but significantly slower (within 100 min) for *S*_*yz*_. We will later use this different evolution speed of *S*_*x*_ and *S*_*yz*_ to draw conclusions concerning the degree of surface localization of the currents *J*_*x*_ and *J*_*yz*_. In contrast to *S*_*x*_ and *S*_*yz*_, measurable changes of the sample reflectance at a wavelength of 790 nm are not observed, thereby ruling optical degradation of our sample out. In addition, we did not observe temporal changes in the signal symmetry, which is discussed in the next section.

### Signal symmetries

In addition to their different amplitude and temporal structure, *S*_*x*_ and *S*_*yz*_ also depend very differently on the sample azimuth *ϕ*. To quantify this behaviour, we measure waveforms *S*_*x*_(*t*, *ϕ*) and *S*_*yz*_(*t*, *ϕ*) for an extended set of *ϕ*-values, example traces of which are shown in Fig. 3a. To reliably extract an average signal amplitude for each *ϕ*, we project the time-domain signal on a suitable reference waveform (see the ‘Methods' section). The resulting signal amplitude as a function of *ϕ* is displayed in Fig. 3b. While both *S*_*x*_ and *S*_*yz*_ exhibit a 120°-periodic component of comparable magnitude, *S*_*yz*_ has a much larger and dominant component independent of *ϕ*.

The three-fold rotational symmetry of the THz signals is fully consistent with the symmetry groups of sample surface and bulk as shown by a detailed analysis of the second-order conductivity tensor (see the ‘Methods' section)^3^. Importantly, it allows us to significantly reduce the large amount of experimental data contained in *S*(*t*, *ϕ*): for a given THz polarization (*x* or *yz*) and pump polarization, each two-dimensional set *S*(*t*, *ϕ*) can be written as a linear combination of just three basis functions (see the ‘Methods' section),


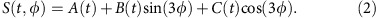


Therefore, three basis signals *A*(*t*), *B*(*t*) and *C*(*t*) fully characterize the entire data set *S*(*t*, *ϕ*). They are, respectively, obtained by projecting *S*(*t*, *ϕ*) onto the mutually orthogonal functions 1, sin(3*ϕ*) and cos(3*ϕ*) (see the ‘Methods' section). Extracted waves are shown in Fig. 3c and Supplementary Fig. 1d for the two THz polarizations and various pump polarizations.

We begin with considering the impact of the pump helicity on the photocurrent. The bottommost curve in Fig. 3c represents the *ϕ*-independent component *A*_*x*_(*t*) of the difference of the signals taken with right-handed (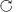) and left-handed (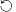) circularly polarized pump light. The amplitude of this waveform is comparable to the noise floor. In other words, a helicity-dependent yet simultaneously *ϕ*-independent THz signal is small and below our detection threshold. This notion is consistent with time-domain raw data (blue versus green trace in Fig. 1b) and the absence of an offset in the *ϕ*-dependence (blue curve of Fig. 3b). An analogous behaviour is observed for THz signals *S*_*yz*_ (see Supplementary Fig. 2). We note that such small magnitude of the pump-helicity-dependent and *ϕ*-independent photocurrent does not contradict the previously reported observation of time-integrated currents^6^ as will be addressed in the ‘Discussion' section.

### Ultrafast photocurrents

**Figure 4:**
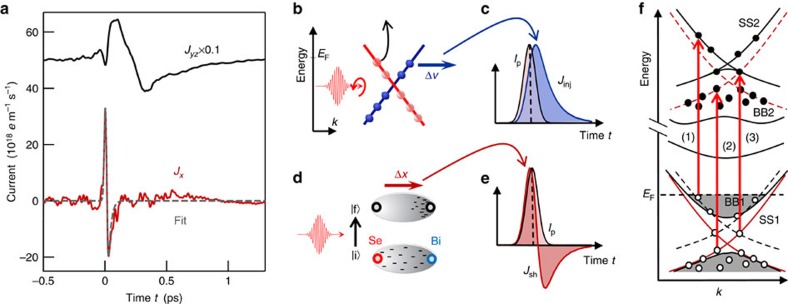
Extracted photocurrents and their assignment. (**a**) Source currents of the two dominant signal components (see Fig. 3). The dynamics of these currents allow us to reveal the origin of the photocurrent. Curves are offset for clarity. (**b**) Example of an injection-type photocurrent. The pump pulse promotes electrons from the Dirac cone into other bands, thereby changing the electron band velocity. An asymmetric depopulation of the Dirac cone and, thus, nonzero net current is achieved by using circularly polarized light^6^. (**c**) Typical shape of the resulting photocurrent *J*_inj_(*t*). Here, *I*_p_(*t*) is the intensity envelope of the laser pulse. (**d**) Scenario of a shift photocurrent arising from an ultrafast transfer of electron density along the Se–Bi bond. (**e**) Typical bipolar temporal shape of the resulting sheet current *J*_sh_(*t*). (**f**) Schematic of the electronic band structure of Bi_2_Se_3_. While BB1/BB2 represent bulk bands below/above the Fermi level *E*_F_, SS1/SS2 refer to surface states. Red arrows indicate three examples of pump-induced optical transitions (photon energy of ∼1.5 eV). To better illustrate the excitation process, thin black/red lines indicate copies of SS1/SS2 shifted upwards/downwards by the pump photon energy. Ground-state occupation is displayed shaded.

Figure 3c leads to another important conclusion of our symmetry analysis: regardless of the pump polarization, all signals *S*_*x*_ and *S*_*yz*_ are, respectively, dominated by just one fast and one slow waveform. We use these signals to extract the underlying source currents (see the ‘Methods' section), which are displayed in Fig. 4a. After an initial onset, both *J*_*x*_ and *J*_*yz*_ change sign, indicating a backflow of charge. Note, however, *J*_*x*_ proceeds much faster than *J*_*yz*_: the rise time from 10 to 90% of the current maximum is 16 and 40 fs for *J*_*x*_ and *J*_*yz*_, respectively. Subsequently, *J*_*x*_ decays with a time constant of 22 fs, while *J*_*yz*_ decays within 700 fs.

To determine the origin of *J*_*x*_ and *J*_*yz*_ based on their ultrafast dynamics, we briefly review known photocurrent generation mechanisms^6^,^8^,^22^,^23^,^24^,^25^,^26^,^27^. In general, optical excitation transfers electrons from initial states 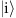 into final states 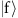 (Fig. 4b), followed by relaxation processes such as scattering into other states, phonon emission and recombination^28^. Photocurrents can arise in both regimes, that is, during the optical transition and during the subsequent relaxation. As our pump photon energy (1.57 eV) is much larger than the Bi_2_Se_3_ band gap, numerous vertical interband transitions are allowed^29^ (Fig. 4f) and expected to outnumber the contribution of phonon- or impurity-assisted nonvertical transitions^30^. In the subsequent relaxation regime, currents can arise from, for instance, scattering by a noncentrosymmetric potential^8^, asymmetric recombination^31^ and carrier acceleration in an intrinsic surface field (drift current)^32^,^33^. In all the cases, inversion symmetry needs to be broken to obtain a macroscopic dipolar net current.

### Drift current

As seen in Fig. 4a, the slow current *J*_*yz*_(*t*) has a rise time (40 fs) significantly slower than the duration of the excitation pulse (≈20 fs). Therefore, it cannot arise from the initial optical transition. In fact, previous works on Bi_2_Se_3_ assigned the slow *J*_*yz*_ component to a carrier drift in the surface field, consistent with the strong dependence of *J*_*yz*_ on the doping level of Bi_2_Se_3_ (refs 9, 10, 11). This notion is further supported by additional observations made in our experiment: first, the initial electron flow is directed toward the sample surface, along the direction of the space–charge field of our effectively n-doped sample. Second, the 40 fs rise time (Fig. 4a) is on the order of the bulk Drude scattering time of our sample (∼18 fs, see the ‘Methods' section) that limits charge acceleration in the surface field.

Following its initial rise, *J*_*yz*_ is found to change sign. Plasma oscillation^34^ of the charge carriers cannot account for this feature because the short Drude scattering time (∼18 fs, see the ‘Methods' section) would strongly attenuate such dynamics in less than 100 fs, in contrast to our observation. We consequently assign the sign change of *J*_*yz*_ to the backflow of charge that accompanies the overall relaxation of the photoexcited system back to the equilibrium state. From photoemission studies^28^, the relaxation of optically excited bulk carriers is known to occur on a timescale of 1 ps, in agreement with the relaxation time of *J*_*yz*_ (see Fig. 4a).

### Shift current

Having assigned the slow, dominant part of current *J*_*yz*_, we now focus on the very fast, sub-100 fs dynamics of the photocurrent *J*_*x*_. Concerning immediate photocurrent generation by an optical transition 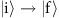, Sipe and colleagues^22^,^35^ used perturbation theory and identified three distinct mechanisms: injection currents, shift currents and optical rectification^22^,^36^. Injection currents *J*_inj_ arise because initial and final state of the perturbed electron have different band velocity. An example is the asymmetric band depopulation scenario^6^ shown in Fig. 4b: a circularly polarized pump excites electrons from the Dirac cone into higher-lying states with different band slope (group velocity). Therefore, for short enough excitation, *J*_inj_ should rise instantaneously to a magnitude that scales with the average velocity change Δ*v* and the density *N* of the excited electrons. In this simplified model, the resulting current is


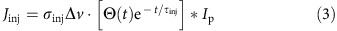


where the initial sheet charge density *σ*_inj_=*eN*Δ*z*_inj_ is proportional to the thickness Δ*z*_inj_ of the emitting sheet, and Θ(*t*) is the unit step function. Note that relaxation processes such as electron scattering are not covered by the theory of ref. 22. We have introduced them phenomenologically by an exponential decay with time constant 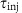. Backflow of electrons is diffusive^37^ and ignored on the short timescales considered here. Finally, the convolution (denoted by ) with the pump intensity envelope *I*_p_(*t*) (normalized to unity) accounts for the shape of the pump pulse, resulting in a current with the typical temporal shape shown in Fig. 4c.

Shift currents^38^, on the other hand, arise when the electron density distribution of the excited state 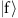 is spatially shifted with respect to 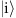 (Fig. 4d). For short excitation, this process leads to a step-like charge displacement Δ*x*_sh_Θ(*t*) whose temporal derivative is proportional to the shift current *J*_sh_. With arguments analogous to the injection case, we obtain


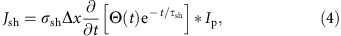


with *σ*_sh_=*eN*Δ*z*_sh_. This model implies *J*_sh_ initially follows the profile of *I*_p_(*t*) and becomes bipolar if the relaxation time 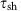 is comparable to or longer than the pump duration (Fig. 4e).

Finally, optical rectification can be understood as a transient, nonresonantly driven charge displacement that follows the intensity envelope of the pump pulse. It arises from all transitions between the initial and final states whose energy difference is different from the incident photon energy of 1.57 eV (ref. 39). To evaluate the relative importance of optical rectification in our experiment, we compared the emitted THz amplitude of our TI sample with that of a pump-transparent ZnTe(110) crystal, which is known for relatively strong optical rectification at the pump photon energy used here^40^. We find that, normalized to the thickness of the emitting crystal, the THz signal *S*_*x*_ from the TI sample is about two orders of magnitude larger than that from ZnTe. Therefore, optical rectification in Bi_2_Se_3_ is expected to make a negligible contribution, and we are left with considering ultrafast injection and shift currents.

Note the characteristic shape of *J*_inj_ and *J*_sh_ is very distinct: unipolar (Fig. 4c) versus bipolar asymmetric (Fig. 4e). Having understood how the temporal shape of a current is intrinsically linked to its origin, we now look for such fingerprints in our data (Fig. 4a). Indeed, we find that the measured photocurrent *J*_*x*_ (Fig. 4a) has bipolar asymmetric temporal shape: the signature of a shift current. In addition, fitting equation (4) to *J*_*x*_ yields excellent agreement (Fig. 4a) for a pump duration of 23 fs, 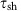
=22 fs and Δ*x*_sh_Δ*z*_sh_≈36 Å^2^. In this procedure, we use the excitation density (*N*=6.9 × 10^24^ m^−3^) as inferred from the absorbed pump fluence (4 μJ cm^−2^), the pump photon energy (1.57 eV) and the pump penetration depth (24 nm at 1/e intensity)^20^. The amplitude spectrum of the calculated *J*_sh_ peaks at 6 THz (not shown), consistent with the peak position of the measured *E*_*x*_ amplitude spectrum (Fig. 1d).

One could argue that the current *J*_*x*_ is not a pure shift current (Fig. 4e) but still contains a component arising from a rapidly decaying injection current (Fig. 4c). We can exclude this possibility based on a symmetry analysis and photocurrent theory work^22^. For example, for linear pump polarization at 45° with respect to the plane of incidence, the resulting current *J*_*x*_ is solely related to the second-order conductivity tensor element *σ*_*xxy*_ (see the ‘Methods' section and Supplementary Note 1). As dictated by symmetry, this element equals *σ*_*xyx*_ and becomes, therefore, real-valued when the photocurrent frequency approaches *ω*=0. From the microscopic analysis of Nastos and Sipe^22^, it follows that this tensor element and the related photocurrent *J*_*x*_ are not due to an injection current, provided that photocurrent frequencies *ω* higher than the current relaxation rate are considered (see the ‘Methods' section).

### Surface localization

Our photocurrent measurements and analysis directly reveal an ultrafast shift current and a drift current in the time domain. It is so far, however, unclear to which extent these currents are localized at the surface. Since the photocurrent observed here is a quadratic nonlinear-optical effect (see inset of Fig. 1c), it only flows in regions where inversion symmetry is locally broken^41^. In our sample, this breaking arises from two perturbations of the inversion-symmetric bulk: (i) the surface, which extends over the first 1 to 2 quintuple layers (∼1 to 2 nm) as indicated by the thickness of surface states^18^ and the depth of surface lattice relaxation,^19^ and (ii) the space–charge field **E**_SC_, which points along the surface normal and extends ∼15 nm into the depth of the sample, as implied by an estimate analogous to ref. 20.

Since the slow component of *J*_*yz*_ is a drift current of photoexcited carriers in the space–charge field^9^,^10^,^11^, it is also localized in a depth of ∼15 nm. In addition, its amplitude changes during sample aging must arise from gradual modifications of **E**_SC_ (Fig. 2). If *J*_*x*_ were also dominated by **E**_SC_, its amplitude should evolve analogously to *J*_*yz*_ following sample cleaving. In contrast, we observe that *J*_*x*_ evolves about five times faster than *J*_*yz*_ (see Fig. 2) such that *J*_*x*_ cannot arise from perturbation (ii) but rather from the remaining perturbation (i). Therefore, the shift current is localized in the first one to two quintuple layers of the Bi_2_Se_3_ surface. The resulting Δ*z*_sh_ of less than 2 nm and the above extracted estimate for Δ*x*_sh_Δ*z*_sh_ imply that the shift distance Δ*x*_sh_ is on the order of 1 Å.

We note that the THz emission spectra exhibit sharp features at 2 and 4 THz (Fig. 1c,d) which coincide with the frequencies of long-wavelength bulk phonon modes at 1.9, 2.1 and 4 THz (ref. 42). While the 1.9 THz mode is infrared-active and can thus absorb pump-generated THz radiation, the 4 THz mode is exclusively Raman active in the inversion-symmetric bulk. Therefore, the presence of the 4 THz feature in the emission spectrum suggests this mode is infrared-activated in the TI surface region where inversion symmetry is locally broken^43^. This effect further underlines the surface sensitivity of THz emission spectroscopy.

## Discussion

Summarizing our results, we have shown that our ultrabroadband THz emission data are fully consistent with the notion that (i) the photocurrent *J*_*x*_ arises from an instantaneous photoinduced shift of charge density by ∼1 Å in an ∼2 nm thick surface region of Bi_2_Se_3_. The displacement relaxes on a very fast timescale of 22 fs. The much slower current *J*_*yz*_ is dominated by a drift current of optically excited carriers in the surface field. (ii) A pump-helicity-dependent and simultaneously azimuth-independent photocurrent is smaller than our detection threshold of 10^18^ *e* m^−1^ s^−1^. This assertion is also valid for other injection-type transport scenarios such as photon-drag currents^44^. It is instructive to discuss these observations and compare them with previous works.

Finding (i) represents the first observation of a surface shift current, which was predicted by Cabellos *et al*.^45^ very recently. We note that we also observe signatures of ultrafast surface shift currents in other TI samples, including thin films^46^ of Bi_2_Se_3_ and Bi_2_Te_3_ (not shown). We emphasize that revealing the time-domain fingerprint of shift currents relies on the 20 fs time resolution of our experiment. Longer pump pulses can easily obscure this signature, even in materials with broken bulk inversion symmetry^36^. The surface shift current is probably the source of the linear photogalvanic surface currents that have been reported previously^6^, but not assigned.

Our results show that the displacement of bound charges occurs in a sheet with thickness Δ*z*_sh_∼2 nm, which is the thickness of the layer where the Dirac states are expected to dominate charge transport^18^. This notion is consistent with reports^19^ showing that only the first quintuple layer exhibits inversion asymmetry on the order of 10%. The shift distance Δ*x*_sh_∼1 Å compares well with reported charge shifts on the order of the bond length (∼3 Å) in noncentrosymmetric semiconductors^38^.

The three-fold azimuthal symmetry of *J*_*x*_ (Fig. 3b) suggests the electron density is displaced along the 120°-ordered p-type Se–Bi bonds^47^. In fact, previous studies have shown that the electron density associated with the Dirac states is known to shift gradually from Se toward Bi atoms when energies below and above the the Dirac point are considered^3^,^47^,^48^. As Bi and Se atoms lie in different layers, the Bi–Se bond forms an angle of about 45° with respect to the sample surface normal. Therefore, the shift current also has a *z*-component with a strength comparable to *J*_*x*_, consistent with the sharp peak present in *J*_*yz*_ at *t*=0 fs (see Fig. 4a and Supplementary Fig. 1d).

In principle, the ultrafast Se–Bi charge transfer can be driven by all kinds of optical transitions involving surface states. Examples of possible transitions induced by the 1.57 eV pump photons are shown in Fig. 4f: (1) surface-to-bulk, (2) bulk-to-surface and (3) surface-to-surface (intercone) transitions. Following excitation, the charge carriers undergo multiple scattering processes, finally resulting in an isotropic distribution. The time constant (22 fs) of this process found here is consistent with other measurements of anisotropy relaxation at the TI surface: ultrafast optical Kerr effect (time constant of ∼25 fs)^49^ and equilibrium transport experiments (substantially smaller than 300 fs, see Supplementary Note 2)^50^,^51^. Note that due to spin-momentum locking at the TI surface, the decay of the anisotropy of the charge-carrier distribution is strongly connected with the decay of the transient surface spin polarization^52^. The timescales of these processes are much shorter than that of the energy relaxation of the surface charge carriers (∼1 ps; ref. 28).

Result (ii), the absence of a pump-helicity-dependent photocurrent, is surprising and imposes significant constraints on the generation mechanism of this current. We first note this result is consistent with the photocurrent magnitudes found by previous electrode-based time-integrating^6^ and picosecond-resolved^13^ measurements. From these works, it follows that under excitation conditions similar to ours, the pump-helicity-dependent photocurrent reaches a peak value that is slightly below the detection threshold 10^18^ *e* m^−1^ s^−1^ of our setup (see Supplementary Note 3).

It is instructive to compare the upper photocurrent limit set by our experiment to a recently suggested microscopic scenario^6^ in which the pump-helicity-dependent photocurrent arises from asymmetric depopulation of the Dirac cone by optical transitions into rapidly decaying bulk states (Fig. 4b). On the basis of this injection-type scenario, we use equation (3) to estimate the initial ballistic sheet-current density as *Nev*_D_Δ*z*_D_, where *v*_D_=0.5 nm fs^−1^ is the band velocity in the Dirac cone^28^, Δ*z*_D_=2 nm the thickness of the Dirac states^18^ and *N* is the bulk excitation density. The resulting magnitude of 10^22^ *e* m^−1^ *s*^−1^ is four orders of magnitude larger than the maximum current measured in our experiment.

Possible reasons why the photocurrent magnitude predicted by this plausible scenario so drastically exceeds the actually measured pump-helicity-dependent photocurrent are as follows. First, matrix elements for bulk–surface optical transitions are much smaller than for bulk–bulk transitions. In other words, light absorption is much more pronounced in the bulk than at the surface, in contrast to the tacit assumption of homogeneous absorptivity made in the estimate above. Second, the band velocity of initial and final state are approximately equal (see equation (3)). Finally, there is a great deal of cancellation when summing over the contributions of all optical transitions. Indeed, as shown theoretically, a zero net current results when only optical transitions within the Dirac cone are considered^53^. Experiments using pump pulses with tunable pump photon energy^54^,^55^ will likely provide more insights into the nature of the pump-helicity-dependent photocurrent, in particular, in terms of intracone and below-gap optical transitions^24^,^53^,^54^.

In conclusion, we have measured the dynamics of ultrafast photocurrents on the surface of the Ca-doped three-dimensional model TI Bi_2_Se_3_ with a time resolution as short as 20 fs. We find that the peak amplitude of pump-helicity-dependent photocurrents is much smaller than predicted by a recent model based on asymmetric optical transitions between Dirac-cone and bulk states^6^. For the first time, we have observed a surface shift photocurrent, which arises from a charge displacement at the TI surface. The fast decay time (22 fs) of this current attests to the rapid loss of charge-carrier surface anisotropy. On a more applied note, the shift current is potentially interesting for ultrafast optical manipulation of the TI surface, thereby ultimately modifying its topological properties^56^. The local electric field generated by the shift current could be used to drive highly spin-polarized THz electric currents at the TI surface. The direction of this secondary current could easily be controlled by the pump-beam polarization, thereby opening up new possibilities to access the TI surface states. Finally, our results highlight broadband THz emission spectroscopy as a novel and highly sensitive probe of surfaces.

## Methods

### Sample details

Single crystals of Ca-doped Bi_2_Se_3_ were grown by the Bridgman–Stockbarger method by pulling a sealed quartz ampoule in a vertical temperature gradient. A fresh surface is obtained by cleaving using adhesive tape. The dimensions of the sample used for THz emission spectroscopy are approximately 2 × 3 × 0.3 mm^3^.

For characterization of the sample by angle-resolved photoelectron spectroscopy (ARPES), the sample was studied directly after cleaving under ultrahigh-vacuum conditions (pressure 2 × 10^−10^ mbar) and, a second time, after exposing the sample to an ambient-pressure atmosphere of N_2_ for a few seconds. ARPES measurements on the N_2_-exposed surface confirm the presence of Dirac surface states with the Fermi energy located roughly 100 meV above the bulk conduction band minimum (see Supplementary Fig. 3). From these data, a conduction-band electron mass of 0.115 bare electron masses is inferred^57^.

Hall measurements^58^ yield a bulk hole density of 1.34 × 10^17^ cm^−3^ and a mobility of 275 cm^2^ V^−1^ s^−1^. Using these values, the effective mass inferred from ARPES data and the Drude formula, we extract a velocity relaxation time of 18 fs in the bulk material.

### Ultrafast THz emission setup

Laser pulses (duration of ≈20 fs, centre wavelength of 790 nm, energy 1 nJ) from a Ti:sapphire oscillator (repetition rate of 80 MHz) are focused onto the sample (beam diameter of 200 μm full-width at half intensity maximum) under 45° angle of incidence, resulting in an average intensity <0.3 kW cm^−2^, well below sample damage threshold. The specularly emitted THz pulse is focused onto an electro-optic crystal in which the THz electric field is detected by broadband electro-optic sampling^40^. We use a (110)-oriented GaP crystal (thickness of 250 μm) owing to its relatively flat and broadband response function^16^. The only exceptions are the measurements of the two-dimensional data set *S*(*t*, *ϕ*) (Fig. 3 and Supplementary Fig. 1), which are sped up by using a (110)-oriented ZnTe crystal (thickness of 300 μm), which exhibits an enhanced detector response at the expense of reduced bandwidth. To calibrate the direction of the measured photocurrent, we use a photoconductive switch as a reference emitter in which the direction of the initial photocurrent burst is along the direction of the external bias field.

Optical wave plates are used to set the polarization state of the pump pulse to linear (with arbitrary rotation angle) or circular. Here, it is essential to avoid any optically birefringent elements (including mirrors) between wave plate and sample. A THz wire-grid polarizer (field extinction ratio of 10^−2^) allows us to measure the *x*- and *yz*-components *E*_*x*_ and *E*_*yz*_ of the THz electric field separately, thereby disentangling current components *J*_*x*_ and *J*_*yz*_, the latter being a linear combination of *J*_*y*_ and *J*_*z*_ (see Fig. 1a and equation (8)). To ensure the electro-optic THz detector has an identical response to *E*_*x*_ and *E*_*yz*_, a wire-grid polarizer with 45° orientation is placed in front of it. Variation of the sample azimuth *ϕ* is performed by attaching the sample to a computer-controlled rotation stage.

### From THz signals to THz fields

To proceed from the measured electro-optic signal *S*(*t*) to the THz electric field **E**(*t*) directly above the sample surface, we note that there is a linear relationship between the two quantities. For example, in the frequency domain, the THz field component *E*_*x*_ and the corresponding signal *S*_*x*_ are connected by the transfer function *h*(*ω*) through the simple multiplication


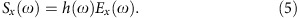


A completely analogous relationship is valid for *E*_*yz*_ and *S*_*yz*_. We measure the transfer function of our setup by using 50 μm thick GaP(110) as a reference emitter placed before the TI sample that has been substituted by an Ag mirror. Details of the shape of the transfer function are shown in Supplementary Fig. 4 and discussed in the Supplementary Note 4.

### From THz fields to THz currents

To finally obtain the source current **J**(*t*) from the THz electric field **E**(*t*) measured directly above the sample surface, we make use of the following generalized Ohm's law^34^:


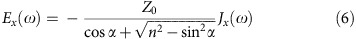



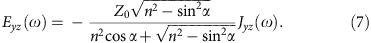


Here, *ω*/2*π* is the THz frequency, *Z*_0_≈377 Ω is the vacuum impedance, *n*(*ω*) is the refractive index of Bi_2_Se_3_ taken from ref. 50, *α*=45° is the angle of incidence, and


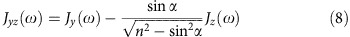


is a weighted sum of the currents flowing along the *y* and *z* directions. For Bi_2_Se_3_ and frequencies above 5 THz, the weighting factor 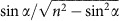 of *J*_*z*_ is on the order of 0.3. The inverse Fourier transformation of the resulting current spectra yields the currents in the time domain (Fig. 4a).

We note that the overall magnitude of the extracted currents is subject to an estimated uncertainty on the order of 3, which arises from the cumulated effects of uncertainties of beam parameters (such as beam diameter and divergence), of the precise optical material properties (in particular, close to phonon frequencies) and from the deviations of the optical and THz beam from a perfect Gaussian profile.

### Waveform mean amplitude

To characterize a complete THz waveform *S*(*t*) by a single mean amplitude 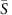, an often-used procedure is to calculate the root-mean square 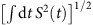. The drawback of this method is that it is based on nonlinear operations, which annihilate phase information and make the identification of additive signal components difficult. Therefore, calculation of a mean amplitude 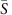 of a given signal should be linear with respect to *S*(*t*). Our solution is to project *S*(*t*) on a suitable and normalized reference waveform *S*_ref_(*t*) by means of the scalar product


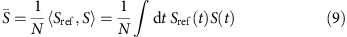


where *N*=〈*S*_ref_, *S*_ref_〉^1/2^ normalizes *S*_ref_/*N* to unity. As required, this operation is linear with respect to *S*(*t*) and, therefore, does not mix up additive signal components.

In the case of our two-dimensional data set *S*(*t*, *ϕ*), we use the most intense signal (with respect to *ϕ*) as reference. This choice is arbitrary, but we checked that other reference waveforms yield qualitatively identical results within our signal-to-noise ratio. According to equations (2) and (9), the basis functions *A*(*t*), *B*(*t*) and *C*(*t*) of the data set *S*(*t*, *ϕ*) are then obtained by multiplying *S*(*t*,*ϕ*) with 1/2*π*, sin(3*ϕ*)/*π* and cos(3*ϕ*)/*π*, respectively, and subsequent integration from *ϕ*=0 to 2*π*.

### Azimuthal symmetry analysis

The inset of Fig. 1c shows that the THz signal grows linearly with the pump power, that is, with the square of the pump field **F**(*t*). Therefore, the resulting current density can phenomenologically be described by the general nonlocal relationship^59^


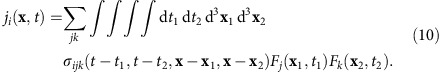


Here, *F*_*j*_ is the *j*th Cartesian component of the pump field, and *σ*_*ijk*_ is a third-rank tensor field describing the quadratic material response. Upon sample rotation described by the matrix *R*_*i*′*i*_, the nonlinear response function transforms according to


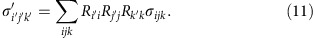


When we focus on rotations about the sample normal by an angle *ϕ*, the matrix elements *R*_*i*′*i*_ are given by linear combinations of the functions 1, cos*ϕ* and sin*ϕ* or, equivalently, 1, exp(i*ϕ*) and exp(−i*ϕ*).

Since the *R*_*i*′*i*_ show up in third order in the response transformation described by equation (11), the transformed 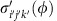 is a third-order mixed polynomial with respect to 1, exp(i*ϕ*) and exp(−i*ϕ*), that is, a linear combination of the terms exp(i*mϕ*) where the integer *m* runs from −3 to +3. From these seven terms, however, only exp(−3i*ϕ*), exp(3i*ϕ*) and 1 remain since our sample is invariant under rotation by 2*π*/3=120° about the surface normal. In other words, 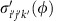 is a linear combination of the functions 1, sin(3*ϕ*) and cos(3*ϕ*).

As seen from equations (1) and (6, 7, 8), the THz field emitted by the current–density distribution **j** is linear with respect to **j** with a proportionality constant that depends on the material only through the linear-optical constants at THz frequencies. Since for our sample these constants are invariant under azimuthal rotations, the *ϕ*-dependence of the THz signal is inherited by that of **j** and, thus, 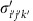.

To summarize, the THz signal depends on the sample azimuth *ϕ* through a linear combination of the three terms 1, sin(3*ϕ*) and cos(3*ϕ*) (see equation (2)). This conclusion is consistent with our experimental observations (see Fig. 3b and Supplementary Fig. 1c).

### Extended symmetry analysis

While the previous subsection focused on rotations about the sample surface normal by an angle *ϕ*, we now also allow for variations of the pump beam's angle of incidence *α* and the polarization state of the pump field. By using the electric-dipole approximation and a Fourier transformation with respect to time, equation (10) yields the photocurrent–density amplitude^59^


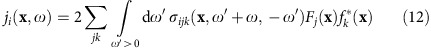


at THz frequency *ω*>0. Here, **F** and **f** are the complex-valued pump-field Fourier amplitudes at frequencies *ω*+*ω*′ and *ω*′, respectively.

The third-rank tensor *σ*_*ijk*_ is nonzero only in regions of broken inversion symmetry, that is, at the surface region of our sample. According to the *C*_3*v*_ symmetry of the sample surface, there are only five independent tensor elements: *η*_1_=*σ*_*xzx*_=*σ*_*yzy*_, *η*_2_=*σ*_*xxz*_=*σ*_*yyz*_, *η*_3_=*σ*_*zxx*_=*σ*_*zyy*_, *η*_4_=*σ*_*yyy*_=−*σ*_*yxx*_=−*σ*_*xxy*_=−*σ*_*xyx*_ and *η*_5_=*σ*_*zzz*_. The other tensor elements are zero^41^.

If the driving field has an angle of incidence *α* and polarization components *F*_*s*_, *f*_*s*_ and *F*_*p*_, *f*_*p*_ along the directions perpendicular and in the plane of incidence, respectively, the resulting photocurrents are given by


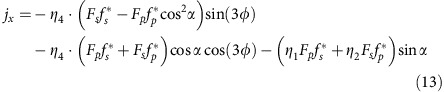



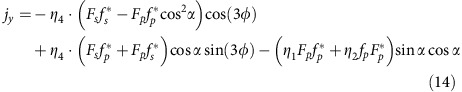



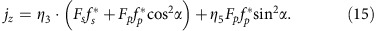


Here, we have omitted the frequency integral and the factor of 2 of equation (12) for brevity. These equations in conjunction with equations (1) and (6, 7, 8) show that the three-fold symmetric current contributes about equally to *S*_*x*_ and *S*_*yz*_, consistent with the observed amplitude variations seen in Supplementary Fig. 1d.

Note the tensor element *η*_4_=−*σ*_*xxy*_=−*σ*_*xyx*_ is symmetric under exchange of the last two indices. Therefore, *η*_4_ becomes purely real-valued when the frequency approaches *ω*=0. On the basis of photocurrent theory work^22^, this property is indicative of a shift current. In contrast, injection currents require *σ*_*ijk*_ to be purely imaginary^22^, which implies sign inversion under exchange of the last two tensor indices. Therefore, any possible injection or pump-helicity-dependent photocurrent is related with *η*_1_ and *η*_2_, and it is independent of the sample azimuth *ϕ*. We note these conclusions are based on the assumption of negligible current relaxation^22^. Therefore, they apply at frequencies *ω* higher than the current relaxation rates 1/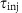 and 1/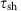.

### Data availability

The data sets generated and analysed during the current study are available from the corresponding authors on reasonable request.

## Additional information

**How to cite this article:** Braun, L. *et al*. Ultrafast photocurrents at the surface of the three-dimensional topological insulator Bi_2_Se_3_. *Nat. Commun.* **7,** 13259 doi: 10.1038/ncomms13259 (2016).

**Publisher's note:** Springer Nature remains neutral with regard to jurisdictional claims in published maps and institutional affiliations.

## Supplementary Material

Supplementary Information Supplementary Figures 1-4, Supplementary Notes 1-4 and Supplementary References
