# Supplementary material for: Ultrafast photocurrents at the surface of the three-dimensional topological insulator Bi2Se3
Source: Nat Commun. 2016 Oct 31;7:13259. doi: 10.1038/ncomms13259 (PMC5095513; doi:10.1038/ncomms13259)
Supplement: Supplementary Information — Supplementary Figures 1-4, Supplementary Notes 1-4 and Supplementary References [file ncomms13259-s1.pdf]

Supplementary Figure 1

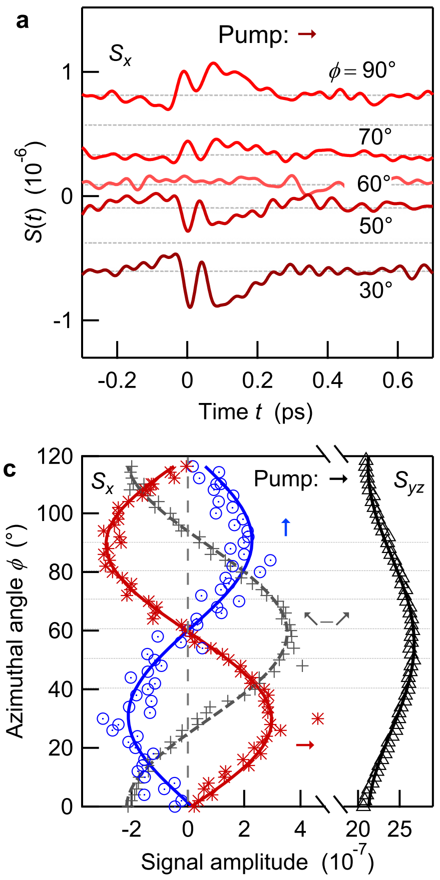

Supplementary Figure 1. Azimuth-dependence and dominant THz signal components. **a**, THz signal waveforms at various sample azimuth angles ϕ and p-polarized pump field. **b**, Additional waveforms for the differential signals $\nwarrow - \nearrow$. **c**, Extracted THz amplitude vs ϕ for various pump-polarization settings ($\rightarrow$, $\uparrow$ and differential signals $\nwarrow - \nearrow$). Dotted lines indicate azimuthal angles of the data shown in **a** and **b**. **d**, Dominant temporal components of signal sets $S_{yz}(t,\phi)$ and $S_x(t,\phi)$ for various pump polarizations, extracted by using Eq. (2).

Supplementary Figure 2

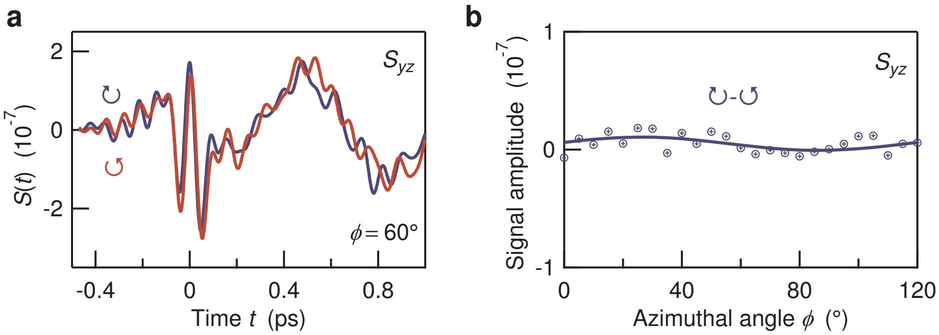

Supplementary Figure 2. Helicity-independence of S_{yz}. **a**, Typical THz signals $S_{yz}(t)$ for circularly polarized pump pulses. **b**, Amplitude of the difference signal (↻ − ↺) for various azimuthal angles. There is no helicity dependence of S_{yz}, consistent with the σ_{ijk} symmetry analysis in the Methods section.

Supplementary Figure 3

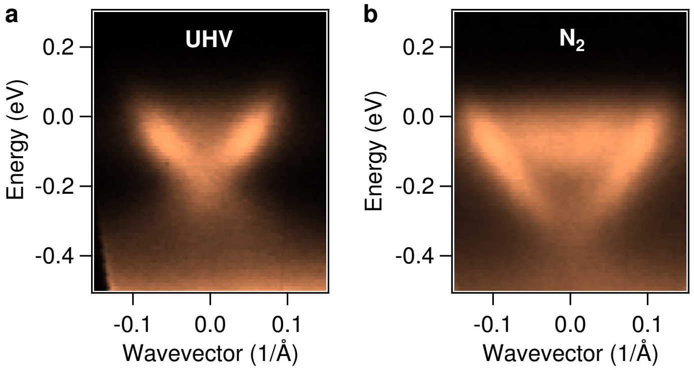

Supplementary Figure 3. Photoemission data of the Ca-doped Bi_2Se_3 sample crystal. **a**, Angle-resolved photoemission data confirm the existence of topological surface states in our sample. Measurements on the pristine surface, obtained directly after cleaving in ultrahigh vacuum, reveal the chemical potential at the lower edge of the conduction band minimum. **b**, Following exposure to 1 bar of N_2 gas, n-type doping shifts the chemical potential into the conduction band, indicating a downward band bending at the surface on the order of 100 meV.

Supplementary Figure 4

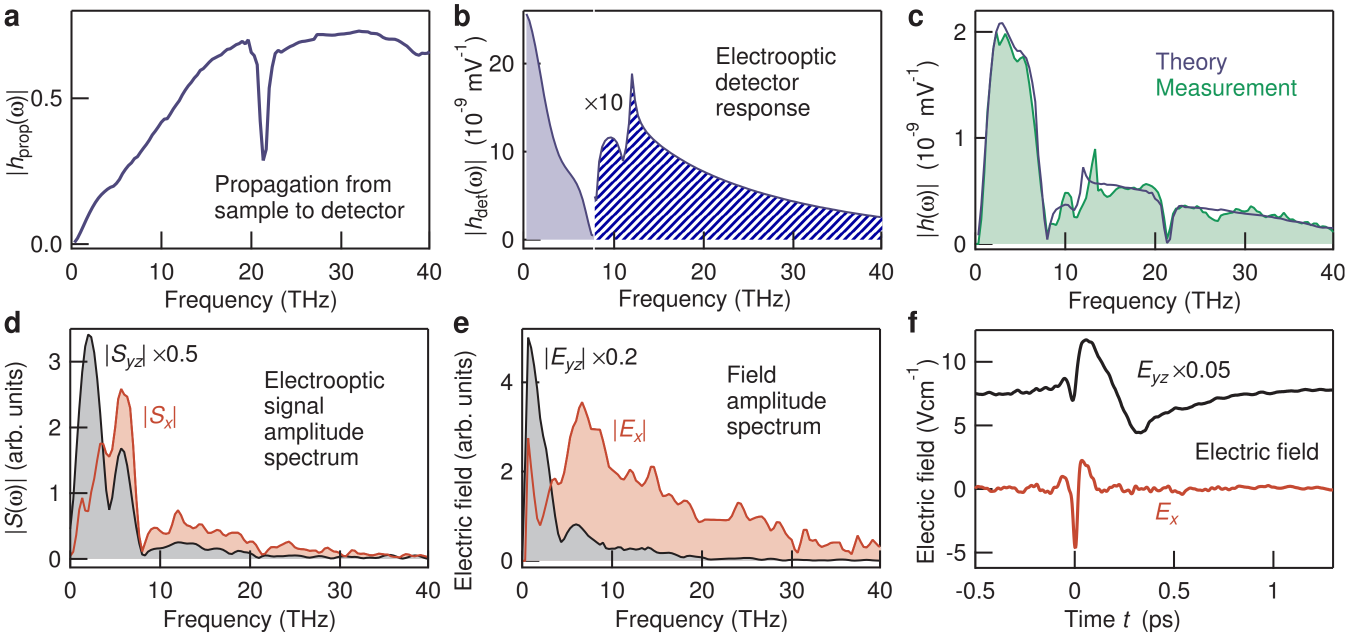

Supplementary Figure 4. From THz signals to THz fields. **a**, Transfer function h_{prop} describing the propagation from the sample to the electrooptic detection crystal. Focusing of the THz pulse leads to the typical high-pass behavior of h_{prop}. The absorption dip at 21 THz arises from the substrate of the wire-grid polarizer used. **b**, Transfer function h_{det} of the electrooptic detection consisting of a 250 μm thick GaP(110) crystal in conjunction with a 20 fs, 790 nm sampling pulse. **c**, Measured and calculated total transfer function $h(\omega) = h_{\mathrm{prop}}(\omega)h_{\mathrm{det}}(\omega)$ of our setup over a broad bandwidth from $\omega/2\pi = 0.3$ to 40 THz. Sharp spectral dips and peaks are related to the Restrahlen band of the GaP detection crystal (see text). **d**, Spectra of typical THz emission signals S_x and S_{yz} clearly exhibit central features of the total transfer function of our spectrometer. **e**, Field spectra obtained by dividing the signal spectra of panel **d** by the transfer function h of panel **c**. **f**, Transient electric field $\mathbf{E}(t)$ of the THz pulse directly after the sample as obtained by an inverse Fourier transformation of $\mathbf{E}(\omega)$. Curves are offset for clarity.

Supplementary Note 1

Signal azimuthal symmetries. The σ_{ijk} symmetry analysis (see Methods) allows us to predict the dependence of the THz signal on the experimental geometry (including sample azimuth and pump polarization). Most notably, all signals exhibit the threefold azimuthal symmetry of the sample surface (see Fig. 3b and Supplementary Fig. 1c). We now discuss features related to the dependence on ϕ and the pump polarization in more detail.

For example, when the y-axis is parallel to an in-plane mirror axis of the $Bi_2Se_3(111)$ surface ($\phi = 0°$ in Fig. 1a), the σ_{ijk} tensor elements of the shift-current signal fulfill $\sigma_{xxy} = \sigma_{xyx}$. Consequently, the pump field amplitude vectors $(E_s, E_p) = (1, 1)$ (i.e. $\nearrow$) and $(1, -1)$ (i.e. $\nwarrow$) make an entirely reversed contribution to J_x, as is also directly apparent from Eqs. (13) and (14). Similar arguments apply when the sample is rotated by 30° (such that the in-plane mirror axis coincides with the x axis) and pump fields $(E_s, E_p) = (1, 0)$ (i.e. $\uparrow$) or $(1, 0)$ (i.e. $\rightarrow$) are used. Finally, at arbitrary ϕ, we obtain a superposition of the effects parallel/perpendicular to the mirror axis.

In Supplementary Fig. 1, we present raw data of the shift current detected along x for (a) p-polarized pump field and (b) the difference signal ($\nwarrow - \nearrow$) for representative azimuthal angles ϕ. As expected, when the sample is rotated by 30°, the shift current signal has maximum/minimum magnitude. After a half-cycle rotation ($\phi = 60°$), the signal arising from a p-polarized pump has reversed sign. In Supplementary Fig. 1c, we plot the amplitude dependence of the same signals (Supplementary Fig. 1a,b) for an extended set of azimuthal angles. The amplitude dependence for s polarization as well as for the S_{yz} signal are also shown.

Having identified the symmetry of the signal (Supplementary Fig. 1c), we use our projection method (see Methods) to extract basis signals $A(t)$, $B(t)$ and $C(t)$ from each two-dimensional data set $S(t, \phi)$ [see Eq. (2) and Supplementary Fig. 1d]. We find that the signal with threefold azimuthal symmetry observed under various pump polarizations and detector orientations derives from only one basis waveform.

Supplementary Note 2

Spin relaxation in Bi_2Se_3**.** The magnitude of optically excited spin-polarized currents depends strongly on the spin relaxation time. In Bi_2Se_3 and at room temperature, optical pump-probe experiments have shown that the optically spin polarization relaxes on a time scale of 100 fs.[1–3]

This value is consistent with equilibrium transport measurements.[4,5] Broadband THz conductivity measurements at 6 K yielded a velocity relaxation time of 1 ps for the surface electrons.[4] Due to spin-momentum locking, this time constant must equal the surface spin-relaxation time. By increasing the sample temperature to 300 K, the DC surface resistivity has been found to increase by a factor 3.[5] Therefore, an upper limit of the surface velocity-relaxation time is given by $\sim$ 1 ps/3 $\sim$ 300 fs. The precise value is probably substantially smaller than 300 fs because the increase of surface resistivity due to an increased carrier scattering rate is counteracted by the increase of carrier density with temperature (which is known to decrease resistivity).

Supplementary Note 3

Current magnitude of the pump-helicity-dependent photocurrent. In our experiment, we find the magnitude of the pump-helicity-dependent and ϕ-independent photocurrent is below the detection threshold $10^{18}\ e\,\mathrm{m}^{-1}\,\mathrm{s}^{-1}$ of our experiment (see Fig. 4a). This result is consistent with the photocurrent magnitudes determined by previous electrode-based time-integrating[1] and time-resolved[6] measurements with a time resolution of $\sim$ 1 ps.

For example, McIver *et al.*[1] obtained a DC photocurrent of $\overline{J} \sim 10^{13}\ e\,\mathrm{m}^{-1}\,\mathrm{s}^{-1}$, normalized to the pump power of our experiment. Assuming the photocurrent flows over a time $\tau_0 \sim 100$ fs and with a repetition rate of $f_{\mathrm{rep}} \sim 100$ MHz, we find the peak current amounts to $J_0 \sim \overline{J}/f_{\mathrm{rep}}\tau_0$, slightly below the detection threshold of our experiment. Note that this comparison has assumed a conservatively small current lifetime τ_0 that is determined by the pump-pulse duration used in Ref. 1. For longer lifetimes, the peak current J_0 would decrease accordingly. A similar consideration can be made for the experiment of Kastl *et al.*,[6] also resulting in peak current values below the detection threshold of our setup.

We note that our straightforward comparison of electrode-based photocurrent measurements[1,6] with our contact-free THz emission approach is justified by the Shockley-Ramo theorem[7].

Supplementary Note 4

From electrooptic signals to THz fields. Supplementary Fig. 4 shows the steps and transfer functions that take us from the detected electrooptic signal to the THz electric field directly after the sample (Supplementary Fig. 4f). The calculated transfer functions[8] account for the effects of THz field propagation (h_{prop}, see Supplementary Fig. 4a) and electrooptic detection (h_{det}, Supplementary Fig. 4b), resulting in a total transfer function h that agrees well with that determined experimentally (Supplementary Fig. 4c).

Supplementary Fig. 4d displays spectra of electrooptic signals S_x and S_{yz}. Spectral features in the range from 8 THz to 15 THz correspond to the Reststrahlen band of the GaP electrooptic detector. The dip at 8 THz arises because electronic and Raman contributions to the electrooptic response of GaP cancel at this frequency. Division of the signal spectra by $h(\omega)$ greatly cancels these features and yields the field spectra E_x and E_{yz} shown in Supplementary Fig. 4e and Fig. 1d. The frequencies of the spectral features at 2 THz and 4 THz agree with Bi_2Se_3 phonon resonances[9]. Below 1 THz, the response decreases sharply to zero because electromagnetic radiation is emitted less efficiently at decreasing frequency.

References

[1] McIver, J. W., Hsieh, D., Steinberg, H., Jarillo-Herrero, P. & Gedik, N. Control over topological insulator photocurrents with light polarization. *Nat. Nanotechnol.* **7**, 96–100 (2012).

[2] Boschini, F. *et al.* Coherent ultrafast spin-dynamics probed in three dimensional topological insulators. *Sci. Rep.* **5**, 15304 (2015).

[3] Wang, M., Qiao, S., Jiang, Z., Luo, S. & Qi, J. Unraveling photoinduced spin dynamics in the topological insulator Bi_2Se_3. *Phys. Rev. Lett.* **116**, 036601 (2016).

[4] Valdés Aguilar, R. *et al.* Terahertz response and colossal Kerr rotation from the surface states of the topological insulator Bi_2Se_3. *Phys. Rev. Lett.* **108**, 087403 (2012).

[5] Bansal, N., Kim, Y. S., Brahlek, M., Edrey, E. & Oh, S. Thickness-independent transport channels in topological insulator Bi_2Se_3 thin films. *Phys. Rev. Lett.* **109**, 116804 (2012).

[6] Kastl, C., Karnetzky, C., Karl, H. & Holleitner, A. W. Ultrafast helicity control of surface currents in topological insulators with near-unity fidelity. *Nat. Commun.* **6** (2015).

[7] Song, J. C. W. & Levitov, L. S. Shockley-Ramo theorem and long-range photocurrent response in gapless materials. *Phys. Rev. B* **90**, 075415 (2014).

[8] Kampfrath, T. *et al.* Terahertz spin current pulses controlled by magnetic heterostructures. *Nat. Nanotechnol.* **8**, 256–260 (2013).

[9] Madelung, O., Rössler, U. & Schulz, M. Bismuth selenide (Bi_2Se_3) phonon dispersion, phonon frequencies, in *Non-Tetrahedrally Bonded Elements and Binary Compounds I*, 1–3 (Landolt-Börnstein - Group III Condensed Matter, Springer Berlin Heidelberg, 1998).
